# Phaneroptic characterization and zoometric indices of Creole goats in the Ayacucho Region, Peru: First step for breeding programs, selection, and conservation

**DOI:** 10.5455/javar.2024.k799

**Published:** 2024-06-22

**Authors:** Walter Palomino-Guerrera, Yeferson Laimes Estrada, David Godoy Padilla, Juancarlos Cruz Luis, Fritz Trillo Zárate

**Affiliations:** 1 Instituto Nacional de Innovación Agraria, Lima, Perú; 2 Escuela de Medicina Veterinaria, Facultad de Ciencias Agrarias, Universidad Nacional de San Cristobal de Huamanga, Ayacucho, Perú

**Keywords:** Creole goats, phaneroptics, typology, zoometric measurements, zoometric indices

## Abstract

**Objective:**

The purpose of this study is to evaluate the phenotypic characteristics and typification of Creole goats in five localities of the Ayacucho region in south-central Peru.

**Materials and Methods:**

Data from 149 goats (25 males and 124 females) were collected, excluding animals under 2 years of age, pregnant, and sick. Seven qualitative characteristics and 11 zometric measurements were evaluated, and then 9 zometric indices were estimated.

**Results:**

In the region, goats with composite colors predominated (76.50%). Additionally, supernumerary (24.20%) and divergent teats (22.60%) were observed in females, while males exhibited a scrotal bifurcation (32.00%). The origin of the flock had a significant effect *(p* < 0.05) on body measurements, as well as on pelvic index, transverse pelvic index, longitudinal pelvic index, compactness index, and load cannon bone index, except for body index (BOI), proportionality index, dactyl thorax index (DTI), and relative cannon bone thickness index (<*p* > 0.05). In the Ayacucho region, there is a predominance of light animals (57.72%) with a significant DTI (<*p* < 0.05) among the populations, including goats with good balance and capable of walking long distances. Furthermore, there are compact animals (47.65%) with a significant BOI (<*p* < 0.05) among the flock populations, which animals are of the biotype of meat. Conversely, the strongest positive correlation (<*r* = 0.89) was discovered between chest girth and body weight.

**Conclusion:**

In the Ayacucho region, there are various creole goat biotypes range from light to very compact heavy goats, with a predominance of meat biotype animals, as well as a marked size dimorphism between localities of origin.

## Introduction

Goat farming is experiencing greater development in various parts of the world, gaining interest for its ability to adapt easily to dietary changes, its resilience to climate change, and its contribution to sustainable plant and soil management [1]. Peru has a goat population of 1,774,523 goat heads, mainly distributed in the highlands and coast, with Piura (317,861 heads), Huancavelica (187,344 heads), and Ayacucho (171,083 heads) having the highest populations [2]. In developing countries, goat farming in rural areas is crucial as it contributes to economic and sociocultural growth and the settlement of populations [3]. Furthermore, goat milk and meat production ensure food safety and economic sustenance for small farmers with limited resources [1,4,5].

The Peruvian Creole goat livestock has a wide genetic diversity that entered the country in colonial times and has evolved over many generations according to the geographical environment where it was settled [6]. Therefore, regional ecotypes are irreplaceable animal zoogenetic resources in their genetic composition, considered a traditional and cultural legacy [7]. In the 1990s, exotic dairy, meat, and dual-purpose breeds, such as Alpine, Saanen, Toggenburg, Oberhasli, and Anglonubian, were introduced into Peru, the latter being the one with the greatest influence on Creole meat goats [8]. However, exotic breeds demand expensive breeding and tend to have difficulty adapting to the rustic way in which most local breeders raise them [1].

There are many sociocultural and political factors that influence the establishment of genetic improvement programs in sustainable goat farming in rural areas [5], where rigorous genotypic and phenotypic characterization studies have not been performed yet [9]. For the sustainable use of genetic variability within an ecosystem, it is crucial to carry out conservation work on ecotypes [1], where the use of zoometric indices helps identify the type of racial group and the zootechnical valuation to predict their productive capacities using morphometric values [10,11]. This tool is fundamental for animal improvement programs as it allows the identification of the local animal biotype [1].

Currently, pure Creole goats are being crossbred with exotic breeds such as Anglonubians. However, the zoogenetic resource of the Creole genotype is essential to establish local breeds adapted to the geographical environment and the breeding system, where the important productive traits derived from exotic breeds and the hardiness to the harsh environment are determined by the Creole goat [1]. In this sense, this study aims to evaluate the phaneroptic characteristics and typification by zoometric indices of the Creole goat in the Ayacucho region, in the southeast of Peru.

## Materials and Methods

### Ethical statement

The owners obtained verbal consent to record the qualitative and quantitative characteristics of the animals. The authors declare that the research work was carried out in accordance with the Ethics Code for animal experiments. (http://ec.europa.eu/environment/chemicals/lab_animals/legislation_en.htm)

### Study area

**Figure 1.**
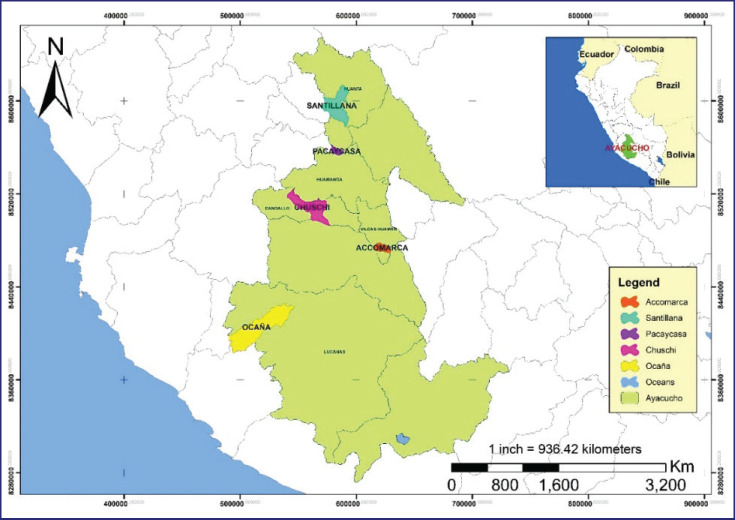
Map of the Ayacucho region showing goat sampling zones.

Five districts in the Ayacucho region, located in southern Peru, were selected between 2,470 and 3,500 m above sea level, considering their representativeness to the goat population each district has (Fig. 1). The districts of Ocaña, Chuschi, Accomarca, Pacaycasa, and Santillana have 2,999, 597, 1,107, 1,231, and 3,092 heads of goats, respectively, according to MINAGRI [2]. These areas have a temperate climate, with a rainy season from December to March and a dry season from May to October. The ambient temperature ranges from 0.1°C in July to 28.7°C in November, with a monthly precipitation of 5.5 to 154.7 mm [12]. Goat farming in Ayacucho is generally extensive, with shrub species predominating as the main food source in the highlands of the steppes. In some districts, transhumance is practiced due to the seasonality of the grazing areas.

### Animals and sample size

**Figure 2.**
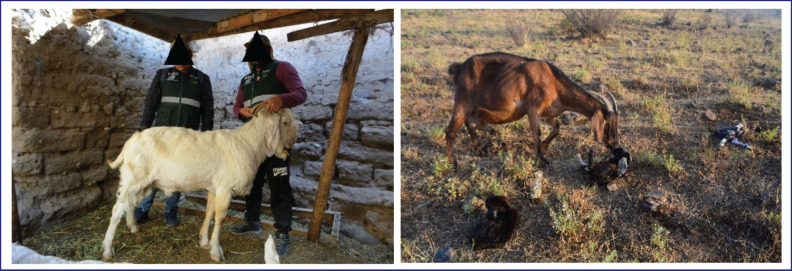
Left: breeding male; right: breeding female, biotype from the Ayacucho Region, Peru.

The animals used were goats with four teeth (approximately 2 years old), randomly selected to avoid bias by biotype preference [13,1]. The sample size was calculated with a sampling error of 10% and a confidence level of 95%. The obtained size was 149 goats, distributed in the districts of Ocaña (n = 33), Chuschi (n = 26), Accomarca (n = 35), Pacaycasa (n = 22), and Santillana (n = 33). Figure 2 shows a male and female breeder biotype from the Ayacucho region.

**Table 1.** Zoometric measurements and their descriptions based on anatomical foundations in goats

Morphometric measurements	Description	References
BW	Weight of the animal in kg	Akounda et al. [13]; Getaneh et al. [14].
WH	Distance in cm from top of the withers to the ground	Saha et al. [15]; Silva-Jarquin et al. [16]
Chest Width (CW)	Distance in cm from the right scapulohumeral joint to the left scapulohumeral joint of the goat	Akounda et al. [13]
RL	Distance in cm from the tip of the hip to the tip of the buttock of the goat.	Saha et al. [15]; Silva-Jarquin et al. [16];
RH	Distance in cm from the ground to the hip of the goat	Akounda et al. [13]; Silva-Jarquin et al. [16]
RW	Distance in cm between the tips of the hips of the goat	Akounda et al. [13]; Silva-Jarquin et al. [16]
BL	Distance in cm from the scapulohumeral joint to the tip of the buttock of the goat buttock	Akounda et al. [13]; Silva-Jarquin et al. [16]
Cannon bone CBP	The girth of the middle metacarpus was measured in cm.	Silva-Jarquin et al. [16]
Paunch girth (PG)	Measurement in cm, the abdominal girth was measured in cm, passing through the first lumbar vertebra and the navel of the goat	Saha et al. [15]
CG	The chest girth was measured, passing through the sternum and the seventh thoracic vertebra	Akounda et al. [13]
BOD	Distance in cm from the umbilical region to the lumbar region of the goat	Saha et al*.* [15]

### Phaneroptic and zoometric measurements

The phaneroptic characteristics evaluated in both males and females included the color of the animal, and the presence of beards, horns, and teats. In females, the number and direction of the teats were assessed, while in males, the presence of scrotal bifurcation was evaluated.

Eleven zoometric measurements were taken according to the anatomical bases shown in Table 1. The morphometric measurements include body weight (BW), wither height (WH), chest width (CW), rump length (RL), rump height (RH), rump width (RW), body length (BL), cannon bone perimeter (CBP), paunch girth (PG), chest girth (CG), and body depth (BOD).

The weight was determined in kilograms using a hanging spring scale with a precision of 0.2 kg [4]. Morphological traits were measured with a plastic measuring tape [17] and a wooden Vernier-type metric ruler known as a zoometric cane.

### Zoometric indices and typology

The following structural indices were estimated: Body index [BOI = Body length (BL / CG) ×100], Pelvic index [PI = rump width (RW) / rump length (RL) ×100], Proportionality index [PRI = (WH / BL) ×100], Dactyl thorac index [DTI = Cannon bone perimeter (CBP / CG) ×100], Transverse pelvic index [TPI = (RW) / wither height (WH) ×100], Longitudinal pelvic index [LPI = (RL / WH) ×100], Compactness index [COI = body weight (BW / WH) / 100], Relative thickness index of Cannon bone [RCTI = (CBP / WH) ×100], and load Cannon bone index [LCI = (CBP / BW) ×100] [17,14].

In addition, goats were typified according to the DTI value as light (<10.5), intermediate (<10.8), light meat (<11), and heavy meat (>11) [14,18], and by the BOI index as brevilinear (<85), mesolinear (>86 and <88), and longilinear (>90) [19,16].

### Statistical analysis

The data were analyzed using R-Statistical software version 4.3.1. Frequency analysis, contingency tables, and bar graph representations were used to evaluate phaneroptic variables. For morphometric variables and indices, descriptive statistics were used to estimate the mean, confidence interval, coefficient of variation (CV), and standard deviation according to the origin and typology of the animal. Pearson’s correlation coefficient (<*r*) was estimated between weight and zoometric measurements [4]. Additionally, the effects of origin and animal typology on zoometric indices were evaluated by analysis of variance (ANOVA) using a univariate linear model, where means comparison was performed using the Tukey test [14,20]. The model for analyzing structural indices was as follows:

**Figure 3.**
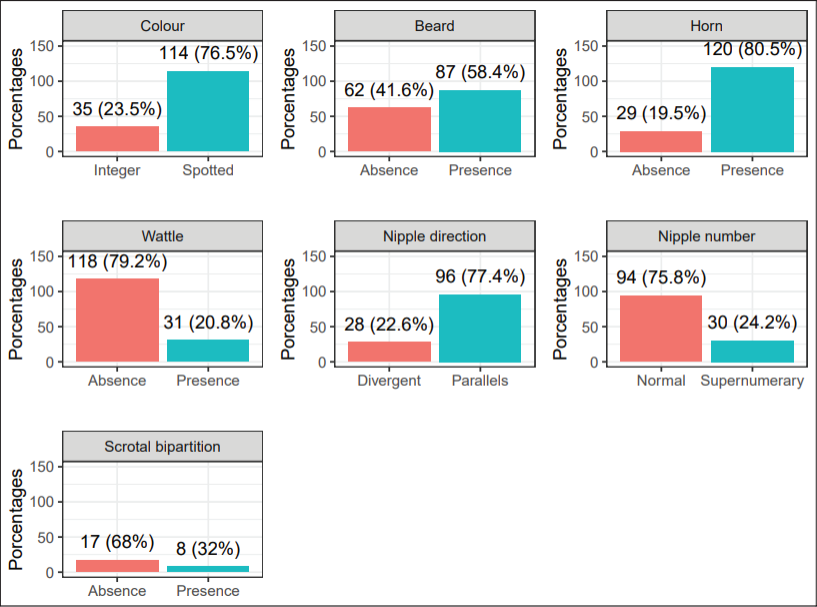
Phaneroptic traits of creole goats from five districts in the Ayacucho Region, Peru.

**Table 2.** Mean and SE of zoometric measurements of Creole goats by district in the Ayacucho region, Peru.

Zoometric Measurements	Ocaña	Chuschi	Accomarca	Pacaycasa	Santillana	General		*p*-value
	Mean ± SE	Mean ± SE	Mean ± SE	Mean ± SE	Mean ± SE	Mean ± SE	VC	
BW	56.11 (1.61)^a^	47.34 (1.83)^b^	36.07(1.95)^c^	40.16 (2.23)^bc^	46.37 (1.47)^b^	45.30 (0.99)	26.60	<0.001
WH	74.00 (0.71)^a^	72.04 (1.01)^ab^	65.87 (0.97)^c^	64.98 (1.17)^c^	68.65 (0.87)^bc^	69.22 (0.50)	8.82	<0.001
CW	19.87(0.31)^a^	20.17 (0.31)^a^	16.47 (0.31)^c^	17.27 (0.40)^bc^	18.49 (0.28)^b^	18.44 (0.18)	12.25	<0.001
RL	24.17 (0.38)^a^	22.89 (0.30)^ab^	21.83 (0.34)^b^	22.73 (0.44)^ab^	22.41 (0.28)^b^	22.79 (0.17)	8.93	<0.001
RH	75.80 (0.82)^a^	74.26 (1.07)^ab^	68.13 (0.93)^c^	69.02 (1.40)^c^	71.65 (0.85)^bc^	71.8 (0.50)	8.47	<0.001
RW	17.17 (0.20)^a^	17.13 (0.26)^a^	13.97 (0.19)^c^	14.98 (0.25)^b^	15.46 (0.21)^b^	15.71 (0.14)	11.03	<0.001
BL	74.40 (1.41)^a^	71.56 (0.90)^a^	66.04 (1.08)^b^	70.00 (1.38)^ab^	70.79 (0.93)^a^	70.47 (0.56)	9.71	<0.001
CBP	9.49 (0.17)^a^	9.09 (0.16)^ab^	8.09 (0.15)^c^	8.45 (0.23)^bc^	8.53 (0.15)^bc^	8.72 (0.08)	11.88	<0.001
PG	98.11 (1.29)^a^	96.63 (1.72)^ab^	83.96 (1.37)^d^	89.77 (1.53)^c^	92.64 (0.86)^bc^	92.07 (0.74)	9.77	<0.001
CG	88.56 (0.94)^a^	85.93 (1.32)^ab^	77.04 (1.08)^d^	80.16 (1.50)^cd^	83.64 (0.78)^bc^	83.05 (0.60)	8.75	<0.001
BOD	37.54 (0.56)^a^	37.07 (0.78)^ab^	31.97 (0.42)^c^	34.43 (0.86)^bc^	36.24 (0.69)^ab^	35.4 (0.33)	11.52	<0.001

a, b, c, Means in the same row with different superscripts indicate significant difference (*p* < 0.05). BW: body weight; WH: wither height; CW: chest width; RL: rump length; RW: rump height; RW: rump width; BL: body length; CBP: perimeter of cannon bone perimeter; PG: paunch girth; CG: chest girth; BOD: body depth; SE: standard errors; CV: coefficient of variation; Ns: non-significant (*p* > 0.05; ***p ≤ 0.001; **p ≤ 0.01; *p ≤ 0.05).

Yij = μ + Bi + εij

where,

Yij = body measurements and zoometric indices.

μ = mean.

Bi = i-th effect of origin or animal typology (i = 1, 2, 3).

εij = residual.

## Results

### Phaneroptic characteristics

The frequency of phaneroptic characteristics of Creole goats in the Ayacucho region is shown in Figure 3. The predominant traits among the evaluated goats (25 males and 124 females) were a composite coat of two or more colors (76.5%), beard (57%), horns (80.5%), and absence of wattles (79.2%). Among females, goats with parallel teats (77.4%) and normal (75.8%, two teats) were found, while the majority of males had a scrotal bifurcation (68%).

### Morphometric analysis and zoometric indices

Zoometric measurements of Creole goats in the five districts are shown in Table 2. Significant differences can be observed between the measurements obtained in the different districts (<*p* < 0.05). The goats of Ocaña were larger than those of the other districts, except for chest width (CW), while the goats of the Accomarca district were the smallest.

The values of the zoometric indices are shown in Table 3. The PI, PRI, TPI, LPI, BOI, and LCI indices had significant differences between districts *(p* < 0.05), whereas for BOI, DTI, and RCTI, no differences were found *(p* > 0.05).

Biotype grouping was performed based on the DTI zoometric index, where light goats predominated at 57.12% and meat-heavy goats at 22.15%. The light-meat goats showed a lower variation in DTI (0.59%) and a higher variation in LCI (26.77%) (Table 4). Groups of goats with light, intermediate, light, and heavy meat affected BOI, DTI, TPI, RCTI, and LCI (<*p* < 0.05), but no evidence was found for the PI, PRI, LPI, and COI indices (<*p* > 0.05). Regarding the group, heavy meat goats were superior in the BOI, PI, PRI, DTI, RCTI, and LCI indices compared to the other indices, while light meat goats were superior in the TPI, LPI, and COI indices.

**Figure 4.**
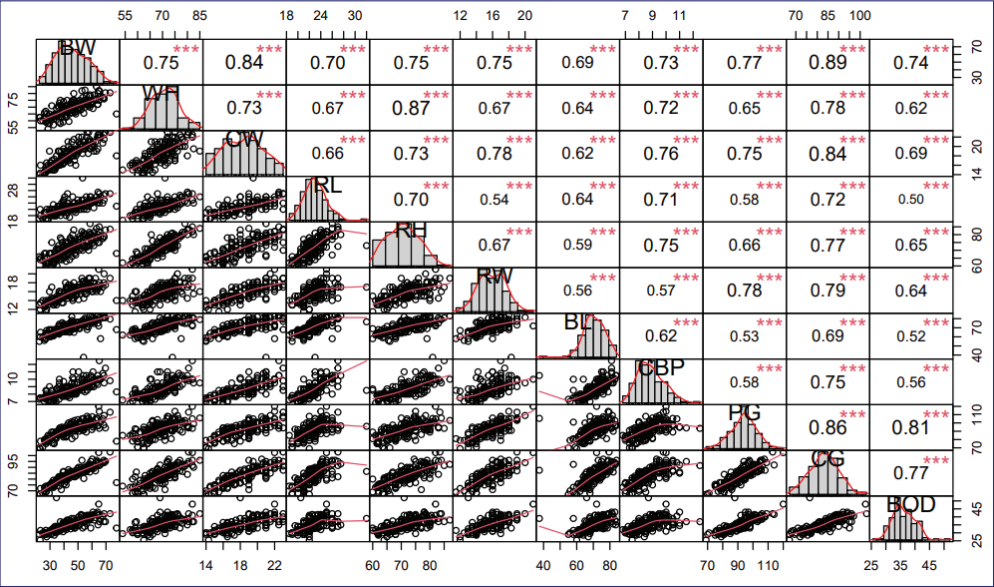
Correlations between zoometric measurements of Creole goats from five districts in the Ayacucho region, Peru. BW: body weight, WH: wither height; CW: chest width; RL: rump length; RW: rump height; RW: rump width; BL: body length; CBP: cannon bone perimeter; PG: paunch girth; CG: chest girth BOD: body depth; (***p ≤ 0.001).

Likewise, biotype grouping was carried out based on BOI, where brevilinear animals predominated (47.65%). The BOI index showed a lower variation (1.43%) in longilinear goats, while the COI index had a higher variation (24.43%) in mesolinear goats (Table 5). The BOI, PRI, and DTI indices had significant differences (<*p* < 0.05) among the biotype groups, while no differences were observed for the PI, PI, LPI, COI, RCTI, and LCI indices (<*p* > 0.05). Brevilinear animals exhibited higher PI, PRI, and TPI values compared to other groups, while BOI, PRI, and LCI were higher in longilinear goats and COI in mesolinear goats. LPI and COI indices were found to be similar in all groups. In the same way, the correlations between the zoometric measurements ranged between 0.50 and 0.89, which are positive. The highest correlation was found between BW and CG (<*r =* 0.89), and the lowest correlation was between RL and body depth (BOD) (<*r =* 0.50) (Fig. 4).

**Table 3.** Mean, SE and CV of zoometric indices for native goat livestock according to districts in the Ayacucho region, Peru.

Zoometric indices	Ocaña		Chuschi		Accomarca		Pacaycasa		Santillana		*p*-value
	Mean ± SE	VC	Mean ± SE	VC	Mean ± SE	VC	Mean ± SE	VC	Mean ± SE	VC	
BOI	84.10 (1.49)^a^	4.48	83.58 (1.18)^a^	7.36	85.70 (0.65)^a^	10.04	87.41 (0.98)^a^	5.25	84.71 (0.95)^a^	6.44	0.174
PI	71.39 (1.08)^ab^	8.55	74.93 (0.89)^a^	6.20	64.32 (0.93)^d^	8.57	66.10 (0.90)^cd^	6.36	69.16 (0.95)^bc^	7.90	<0.001
PRI	101.08 (3.17)^a^	5.99	100.79 (1.14)^ab^	5.87	100.00 (1.01)^ab^	17.74	93.05 (1.20)^b^	6.06	97.32 (1.49)^ab^	8.79	0.0343
DTI	10.72 (0.16)^a^	6.58	10.61 (0.17)^a^	8.51	10.50 (0.12)^a^	8.25	10.52 (0.14)^a^	6.46	10.19 (0.14)^a^	8.06	0.111
TPI	23.23 (0.26)^ab^	7.84	23.83 (0.32)^a^	6.95	21.29 (0.28)^c^	6.32	23.13 (0.38)^ab^	7.65	22.58 (0.31)^b^	7.85	<0.001
LPI	32.68 (0.46)^b^	7.28	31.81 (0.28)^b^	4.52	33.21 (0.41)^b^	8.03	35.02 (0.47)^a^	6.27	32.71 (0.36)^b^	6.39	<0.001
COI	0.008 (0,000)^a^	27.81	0.007 (0.000)^b^	15.80	0.005 (0.000)^c^	12.86	0,006 (0,000)^bc^	19.95	0.007 (0.000)^b^	14.62	<0.001
RCTI	12.81 (0.15)^a^	7.43	12.65 (0.22)^a^	9.19	12.29 (0.15)^a^	6.59	12.98 (0.21)^a^	7.52	12.45 (0.20)^a^	9.37	0.0822
LCI	17.19 (0.38)^d^	21.08	19.92 (0.81)^cb^	21.19	23.83 (0.85)^a^	12.43	21.71 (0.67)^ab^	14.53	18.74 (0.43)^cd^	13.30	<0.001

a, b, c, Mean in the same row with different superscripts indicates a significant difference (*p* < 0.05); BOI: body index; PI: pelvic index; PRI: proportionality index; DTI: Dactyl thorax index; TPI: transverse pelvic index; LPI: longitudinal pelvic index; COI: compactness index; RCTI: relative cannon bone thickness index; LCI: cannon bone loading index; SE: standard errors; CV: coefficient of variation; Ns: non-significant (*p* > 0.05; ***p ≤ 0.001; **p ≤ 0.01; *p ≤ 0.05).

**Table 4.** Comparison of zootechnical indices according to conformation grouping (COI) of native goats from the Ayacucho region, Peru.

Body index	Light animals		Intermediate animals		Light meat animals		Heavy meat animals		*p*-value
	Mean ± SE	VC	Mean ± SE	VC	Mean ± SE	VC	Mean ± SE	VC	
BOI	83.99 (0.47)^a^	5.18	86.84 (0.99)^a^	4.96	84.11 (2.40)^a^	9.48	86.90 (1.53)^a^	10.09	0.0488
PI	70.05 (0.63)^a^	8.33	68.33 (1.30)^ab^	8.31	70.47 (2.29)^a^	10.76	66.65 (1.31)^ab^	11.25	0.0565
PRI	98.30 (0.63)^a^	5.92	96.66 (1.43)^a^	6.44	100.43 (4.05)^a^	13.37	100.58 (3.17)^a^	18.10	0.536
DTI	9.96 (0.04)^c^	3.44	10.64 (0.02)^b^	0.80	10.89 (0.02)^b^	0.59	11.70 (0.11)^a^	5.44	<0.001
TPI	22.97 (0.18)^a^	7.47	22.62 (0.40)^ab^	7.68	23.36 (0.61)^a^	8.65	21.96 (0.37)^ab^	9.62	0.040
LPI	32.88 (0.27)^a^	7.60	33.26 (0.56)^a^	7.28	33.31 (0.78)^a^	7.75	33.06 (0.36)^a^	6.23	0.888
COI	0.006 (0.000)^a^	18.46	0.007 (0.000)^a^	25.40	0,007 (0.000)^a^	23.29	0,006 (0.000)^a^	24.59	0.709
RCTI	12.11 (0.07)^c^	5.54	12.74 (0.19)^b^	6.51	13.05 (0.17)^ab^	4.38	13.66 (0.20)^a^	8.57	<0.001
LCI	19.42 (0.39)^b^	18.75	20.36 (0.92)^ab^	19.75	19.95 (1.61)^ab^	26.77	22.47 (0.88)^a^	22.42	0.006
Proportion (%)	57.72		12.75		7.38		22.15		

a, b, c, Mean in the same row with different superscripts indicates a significant difference (*p* < 0.05); BOI: body index; PI: pelvic index; PRI: proportionality index; DTI: Dactyl thorax index; TPI: transverse pelvic index; LPI: longitudinal pelvic index; COI: compactness index; RCTI: relative cannon bone thickness index; LCI: cannon bone loading index; SE: standard errors; CV: coefficient of variation; Ns: non-significant (*p *> 0.05; ***p ≤ 0.001; **p ≤ 0.01; *p ≤ 0.05).

**Table 5.** Comparison of zoometric indices in Creole goats grouped by biotype (BOI) in the Ayacucho region, Peru.

Body index	Brevilinear		Mesolinear		Longilinear		*p*-value
	Media ± SE	VC	Media ± SE	VC	Media ± SE	VC	
BOI	80.78 (0.68)^a^	7.07	86.94 (0.19)^b^	1.43	91.32 (0.40)^c^	2.54	<0.001
PI	69.94 (0.77)^a^	9.27	68.99 (0.89)^a^	8.58	67.47 (1.18)^a^	10.24	0.184
PRI	101.62 (1.61)^a^	13.34	97.41 (0.77)^ab^	5.21	94.52 (0.94)^b^	5.78	0.0026
DTI	10.20 (0.08)^b^	6.52	10.62 (0.13)^a^	8.17	10.97 (0.13)^a^	7.12	<0.001
TPI	23.09 (0.21)^a^	7.51	22.42 (0.24)^a^	7.22	22.34 (0.24)^a^	10.31	0.0686
LPI	33.14 (0.26)^a^	6.65	32.65 (0.43)^a^	8.72	33.17 (0.36)^a^	6.35	0.507
COI	0.006 (0.000)^a^	18.49	0.007 (0.000)^a^	24.43	0.006 (0.000)^a^	20.99	0.224
RCTI	12.56 (0.11)^a^	7.18	12.57 (0.19)^a^	9.89	12.75 (0.17)^a^	7.93	0.663
LCI	20.10 (0.47)^a^	19.75	19.57 (0.65)^a^	21.96	21.46 (0.83)^a^	22.51	0.145
Proportion%	47.65		29.53		22.82		

a, b, c, Mean in the same row with different superscripts indicates a significant difference (*p* < 0.05); BOI, body index; PI, pelvic index; PRI, proportionality index; DTI, Dactyl thorax index; TPI, transverse pelvic index; LPI, longitudinal pelvic index; COI, compactness index; RCTI, cannon bone thickness index; LCI, cannon bone loading index; Ns, non-significant (*p* > 0.05; ***p ≤ 0.001; **p ≤ 0.01; *p ≤ 0.05).

## Discussion

In this study, animals with diverse traits were evaluated. In the five districts, there are goats with a wide variety of hair color compositions, with composite colors being the most prevalent. Similar results have been reported in the Lima region [21], while in other regions of the world, such as Ethiopia and Tanzania, there are typical distinctions where solid colors or combined colors/composite colors predominate [20,4]. The color of the hair is influenced by the adaptability developed by goats in a specific agroecosystem [4]. For example, in warm environments, light-colored goats are predominant, whereas in cold climates, dark-colored goats are more common. One study suggests that light-colored goats are characterized by a better productive capacity as they tolerate thermal stress better [18], although Hagan et al. [22] mention that light-haired goats lose heat, which affects weight gain. This study reveals a predominance of herds with beards, horns, and no wattles, which agrees with the results of Oyolo [21] in the Lima region but is superior to goats from Ghana and Ethiopia [22,4]. These qualitative traits have functions of self-regulation and defense and are related to expressions of fertility and productivity [4]. Divergent and supernumerary teats are present in about 22% and 24% of females, respectively. The genetic frequency for supernumerary teats is 0.34 [23], predisposing lactating goats to mastitis as this trait affects milking efficiency, causing teat lesions [24]. 32% of males exhibit scrotal bifurcation, which could be related to better spermatic parameters [25].

The existence of a genetic correlation means that qualitative traits influence quantitative traits [6]. Estimating morphometric parameters in goats is very important for establishing genetic improvement programs [13], setting breed standards [7], understanding interspecific variations between populations [26], and also proposing strategies for the conservation of genetic resources, such as a gene bank, for their sustainable utilization [1]. The BW of the evaluated goats ranges from 36 to 56 kg, which is lower than that reported by Oyolo [21] in Lima, who found values between 62 and 80 kg. However, it is higher than the BW of goats both in Tanzania, where it ranges between 24 and 32 kg [20], and in Ethiopia, where it is between 22.9 and 26.4 kg [4]. However, the height of the WH ranges from 64.98 to 74.00 cm, which, according to Devendra and Burns [27], classifies them as medium to large goats. These results are similar to the reports by Maksimović et al. [7] in Serbia but are lower than the goats evaluated in Lima [21], and higher than the native goats of Tanzania [20], Ethiopia [4], and Burkina Faso [13]. Regarding other zoometric measurements, the findings were similar except for CW, which was similar between the cited studies. These variations between populations are influenced by genetic and environmental factors [6] and the indiscriminate use of genetic material [13]. However, the variation was low within the evaluated herds except for BW, which had a high variation (26.60%), possibly due to the selection practiced by the producers during management [20].

Creole goats do not have a defined productive orientation; however, in this study, there were variations between groups in most zoometric indices, while the average values of the BOI (83.58–87.41) were similar across districts. These results are slightly lower than those of goats (89.96) from Ethiopia [14,28] and similar to goats (86–87) from Indonesia [17,11], Cuba [9], and Gamo Gofa [29]. From the BOI obtained, it could be deduced that the Creole goats of Ayacucho, Peru, were a dual-purpose and meat biotype, where the goats with low zoometric indices stood out [10]. However, DTI (10.50) was similar to that of the goats of Ethiopia [14] and Indonesia [17] but higher than that obtained for the goats (9.58) of Cuba [9]. Meanwhile, the PRI (93.05 to 101.08) was lower than that of goats (102.08–105.80) from Ethiopia [14] but higher than that of goats (96.81–97.55) from Indonesia [17], considering that lower values correspond to meat biotype animals [23]. The PI, TPI, LPI, COI, RCTI, and LCI indices were similar to those obtained for goats from Indonesia [25] and higher than those for goats from Ethiopia [14]. Goats with higher values of PI, TPI, and LPI were oriented towards the meat biotype with good maternal and reproductive ability [9,16]; the same could be said for COI, which defines the goat of the Ayacucho region as dual purpose [30].

Phenotypic typification in livestock herds is a practice carried out to determine their productive orientation. However, in Creole goats, it is still uncertain due to the genetic diversity resulting from multiple crosses over many generations. Hence, they are not recognized as a breed [27]. In this study, most goats are light (57.72%; < 10.5), which, according to Khargharia et al. [32], are goats of dairy to dual purpose biotype, and if it were < 10, it would be considered a purely dairy biotype [30]. Since RCTI indicates a good balance and biotype of the animal, goats with high RCTI values have a good balance, which guarantees grazing at large distances without joint problems [19]. Brevilinear goats are of the meat biotype with greater muscle mass and a rectangular shape, while longilinear goats are considered of the dairy biotype [30]. In this study, 47.65% of goats are of the meat biotype, which would be the result of ecological influence, geographical location [29], and the practice of selecting herds according to productive predictions made by producers for generations.

The correlations between BW and zoometric measurements were positive, ranging from 0.50 to 0.89. The highest correlation was found between BW and CG (<*r =* 0.89), while the lowest was found between RL and BOD (<*r =* 0.50), which agrees with Sheriff’s reports [4] in Ethiopia and Depison et al. [17] in Indonesia. Meanwhile, Silva-Jarquin et al. [4] reported completely different results, where the highest correlation was found between rump height and RW (<*r =* 0.59) and BW and CG (<*r =* 0.57). The high correlation between BW and zoometric measurements predicts harmonious growth and good conformation of body structure. The selection of some traits would influence others as long as there is a high correlation, in addition to being a fundamental basis for establishing genetic improvement programs [4].

## Conclusion

The goats of the flocks studied in the Ayacucho region exhibit a wide variety of phenotypic traits, with composite colors, beards, horns, and parallel and normal teats among them. Morphostructure heterogeneity is observed among the goat populations due to adaptation characteristics in each district According to the zoometric indices of productive interest, the goats in the herds are light to heavy with elongated, compact, and well-balanced bodies, oriented towards dual-purpose goat biotypes capable of producing milk and meat and easily walking over long distances. Clearly, the expressed qualities are the result of environmental factors, adaptability, and reproductive practices that have been performed for many decades, becoming a potential zoogenetic resource that requires conservation strategies for subsequent use in the genetic improvement of the herd through the selection of the qualities found.

## List of abbreviations

ANOVA, variance analysis; BL, body length; BOD, body depth; BOI, body index; BW, body weight; CBP, cannon bone perimeter; CG, girth of the chest; COI, compactness index; CV, coefficient of variation; CW, chest width; DTI: Dactyl thorax index; LCI, cannon bone loading index; LPI, longitudinal pelvic index; Ns, non-significant; PG, pillar girth; PI, pelvic index; PRI, proportionality index; RCTI, relative cannon bone thickness index; RL, rump length; RW, rump height; RW, rump width; SE, standard errors; TPI, transverse pelvic index; WH, wither height.
